# Fluorescent Deferoxamine Complexes of Cu(II) and Zr(IV): Insights in the Development of Dual Imaging Probes

**DOI:** 10.1002/chem.202501203

**Published:** 2025-05-27

**Authors:** Yschtar Tecla Simonini Steiner, Virginia Zizi, Monica Mangoni, Patrizia Nardini, Daniele Bani, Andrea Bencini, Antonio Bianchi, Matteo Savastano, Giammarco Maria Romano

**Affiliations:** ^1^ Department of Chemistry “Ugo Schiff” University of Florence Via della Lastruccia 3 50019 Sesto Fiorentino Italy; ^2^ Imaging Platform, Department of Experimental & Clinical Medicine University of Florence Viale G.Pieraccini 6 50139 Florence Italy; ^3^ Radiotherapy Unit, Department of Experimental & Clinical Biomedical Sciences University of Florence Largo Brambilla 3 50134 Florence Italy; ^4^ Department for the Promotion of Human Science and Quality of Life University San Raffaele Roma Via di Val Cannuta 247 00166 Rome Italy

**Keywords:** Cu(II) complexes, deferoxamine, fluorescent PET probes, optical/PET dual mode imaging, Zr(IV) complexes

## Abstract

Deferoxamine (DFO) is widely regarded as benchmark chelator for ^89^Zr(IV), a commonly used PET (positron emission tomography) tracer. We have introduced a novel fluorescent coumarin derivative of DFO (DFOKC), characterized by chelating unit and fluorophore covalently linked via a lysine molecule. This design introduces a free primary amine group, which, in perspective, can be functionalized with biological vectors, potentially improving tumor tissue selectivity. Its acid‐base and metal coordination properties toward Cu(II) and Zr(IV) ions were thoroughly characterized using UV‐Vis and fluorescence emission spectroscopy. DFOKC strongly coordinates both metal ions, forming somewhat more stable complexes than DFO, while retaining fluorescence emission, thus enabling dual‐mode optical and PET imaging. Biodistribution assays conducted on NIH‐3T3 fibroblasts, and MDA‐MB‐231 mammary adenocarcinoma cell lines demonstrated that the presence of primary amine groups favors Zr‐DFOKC complex cell internalization via pinocytosis compared to the parent molecule DFOC, in which the fluorophore is linked to the amine group of DFO. Furthermore, crystal violet and MTT assays revealed no cytotoxic effects or mitochondrial impairment, even at concentrations higher than those typically used for radio‐diagnostic applications. These results strongly support the potential of DFOKC as a versatile and promising tool for dual imaging, offering significant advantages in molecular imaging.

## Graphical abstract

The insertion of a fluorescent coumarin unit by using a lysine‐based linker on deferoxamine leads to a chelating agent (DFOKC), whose Cu(II) and Zr(IV) complexes can be used for both optical imaging and positron emission tomography. DFOKC shows increased binding ability for Cu(II) and Zr(II) and improved tumor cell internalization via pinocytosis.

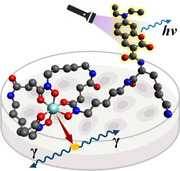

## 1 Introduction

Radiopharmaceuticals are a topical research subject given their potential as diagnostic and therapeutic tools in medicine.^[^ ^1^ ^]^ In the scope of diagnostics, radiolabeling allows for molecular imaging techniques, like positron emission tomography (PET), where the long‐range β^+^ particles emitted by appropriate radionuclides can be detected with high accuracy to yield a detailed analysis of the target organs,^[^ ^2^ ^]^ pivotal in cardiology, neurology, and, overall, oncology diagnostic methods.^[^ ^3^, ^4^, ^5^, ^6^ ^]^ In the last few years, there has been an increasing interest for PET radiolabels containing positron‐emitting metals.^[^ ^7^ ^]^ While nonmetal radioactive isotopes (e.g., ^14^C, ^15^O, ^18^F, ^131^I) are typically covalently incorporated into organic frameworks, a procedure that requires complex synthetic strategies,^[^ ^3^ ^]^ PET agents containing radiometals generally imply a straightforward and fast labelling process that relies on coordination chemistry, for example binding of a metal cation to a chelating ligand, requiring milder conditions (neutral pH, low temperatures) compared to organic PET radiopharmaceuticals. Furthermore, PET radiometals have a wide range of physical half‐lives, ranging from hours to several days, allowing them to be properly chosen depending on the specific diagnostic requirement.

Developing new and efficient radiopharmaceuticals is challenging due to the influence of several critical factors. Among these, a key role is played by the selected radionuclide and the ligand system responsible for coordinating the radiometal ion within a stable complex. An ideal chelator should exhibit high radiometal binding efficiency, thermodynamic stability of the resulting complexes, and sufficient kinetic inertness to minimize its in vivo dissociation, thus preventing the release of toxic metals.^[^ ^8^, ^9^, ^10^ ^]^ In recent years, deferoxamine (DFO) as a chelating moiety began to hold a pivotal role in the design of imaging probes. This bacterial siderophore is made up of a linear chain ligand bearing three hydroxamic domains capable of strongly binding hard metal cations, which show great affinity toward oxygen donors.^[^ ^11^, ^12^, ^13^, ^14^ ^]^ DFO's high affinity toward Fe(III) ions, for example, has been extensively used in FDA/EMA‐approved iron chelation therapies, whose toxicological and pharmacological patterns are well established.^[^ ^15^ ^]^

Detailed characterization of DFO complexes with other trivalent metals (Co, Al, Ga),^[^ ^16^, ^17^, ^18^ ^]^ as well as with bivalent ions (Sn, Cd, Ni) are also widely reported, allowing to consider DFO a drug of choice in treating metal poisoning.^[^ ^19^, ^20^ ^]^ In the case of first transition row divalent metal ions, their binding affinity for DFO is generally lower than Fe(III). However, very strong complexes have been observed with Cu(II), including binuclear species.^[^ ^15^ ^]^ Exploiting the stability of DFO complexes with copper and its well‐established coordination chemistry, ^64^Cu (*T*_1/2_ = 12.7 hours) has been the first DFO‐bound radiometal to be investigated for PET imaging.^[^ ^21^, ^22^, ^23^ ^]^ More recently, attention has been paid to the complexes with ^89^Zr. In fact, the ^89^Zr nuclide has a 78.41 hours half‐life (*T*_1/2_) and a 22.3% positron intensity (I(β^+^)), which make it suitable for PET imaging particularly for studying the kinetics of biologically active molecules with relatively long plasma half‐lives.^[^ ^24^, ^25^ ^]^

As highlighted in previous studies,^[^ ^26^, ^27^ ^]^ hydroxamate groups have long been recognized as effective ligands for zirconium ions, exhibiting strong oxophilic and hard acid tendencies.^[^ ^28^ ^]^ In particular, DFO is one of the most studied and widely used chelators for ⁸⁹Zr(IV) in PET and immuno‐PET applications.^[^ ^29^, ^30^, ^31^, ^32^, ^33^, ^34^ ^]^ Nevertheless, only a few studies exist on the thermodynamic stability and complex formation equilibria in a DFO‐Zr(IV) solution.^[^ ^35^, ^36^, ^37^ ^]^ In the absence of a crystal structure to determine and characterize the coordination properties of Zr(IV), Guerard et al.^[^ ^27^ ^]^ evaluated crystallographic data from a structure of a Zr(IV) complex with simpler hydroxamic acid as analogue models. As expected, when the Zr(IV) charge is neutralized by 4 methyl‐hydroxamates, a square antiprism octa‐coordinated complex is formed.^[^ ^28^ ^]^ Moreover, theoretical calculations estimated the absolute and relative formation constants of Zr(IV) with various ligands, including DFO, and confirmed the stability of a structure where the three hydroxamate groups of DFO are coordinated to the zirconium, together with two water molecules, completing the metal's coordination sphere.^[^ ^38^, ^39^ ^]^ Early studies on complex formation using NMR analysis and complexation rates were conducted by Mejis et al.^[^ ^40^ ^]^ In the same study, the stability of the DFO‐Zr complex in human plasma was also investigated, underscoring high stability in vitro with an estimated metal loss of less than 0.2% after 24 hours. Besides the high stability of the zirconium complexes with DFO, further advantages of DFO depend on its terminal primary amine, not involved in metal complexation, suitable for further modifications, such as bioconjugation and/or labelling with fluorescent molecules.^[^ ^41^, ^42^ ^]^ In this context, developing a fluorophore‐tagged DFO that can retain its fluorescence even when bound to the metal to achieve a bimodal PET/optical imaging probe can be appealing, and this is the strategy we adopted. Holland has recently explored both a similar and an alternative way, that is, tagging the protein moiety of the radiopharmaceutical independently with fluorescent tags and PET active metal complexes.^[^ ^43^ ^]^ Whatever the strategy, these attempts aim to simplify the synthesis of multimodal imaging probes.

**Figure 1:**
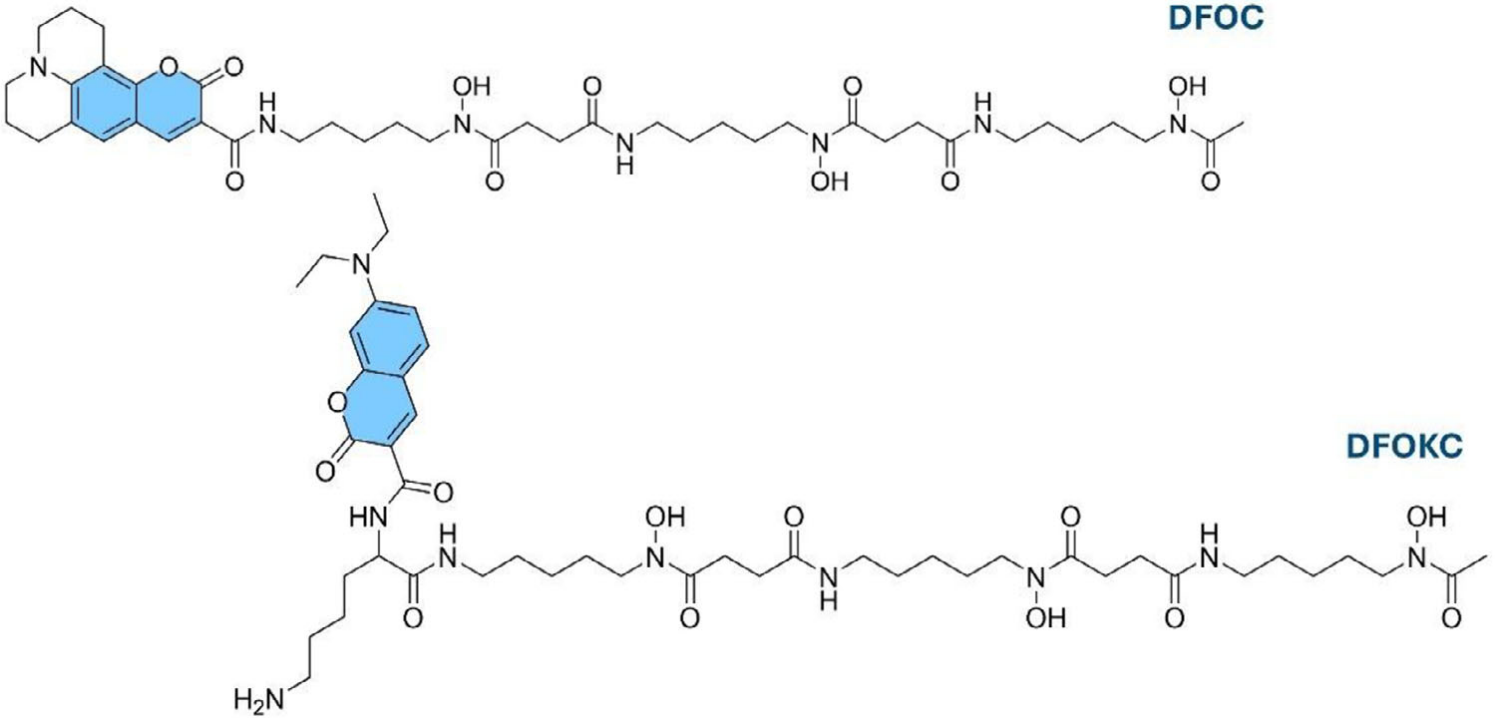
Drawing of DFOC and DFOKC fluorescent ligands.

In previous work, we succeeded in synthesizing a coumarin‐tagged DFO (DFOC, Figure 1) bimodal probe, and we assessed the retention of protonation and coordination properties of hydroxamic groups of the DFO ligand toward Cu(II) and Zr(IV), as well as the fluorescence emission of coumarin.^[^ ^32^ ^]^ In the present study, we have examined the chemical and biological properties of novel Zr(IV) and Cu(II) chelates with a new ligand, termed DFOKC, made up of a DFOC modified by the addition of a lysine (Lys) residue linked to the coumarin moiety, but still bearing a free terminal amine. This new ligand has been designed as an enhanced DFO, which maintains the favorable binding properties of the parent molecule added with a primary amine group aimed to favor bioconjugation, while also incorporating the same fluorophore. Considering its Zr(IV) complex, the presence of the primary amine group should also improve the functional activity of DFO from both a chemical and biological point of view. This includes protonation of the complex in solution and increase in its positive charge. In turn, a higher positive charge should favor the interaction with the negatively charged surface of the cell plasma membrane. On this background, the purpose of the present work was to investigate the chemical coordination and biological properties of the Cu(II) and Zr(IV) complexes with the coumarin‐containing DFOKC ligand in comparison with the parental DFOC chelates, to verify whether these improved compounds can also be developed as suitable bimodal imaging agents.

## 2 Results and Discussion

### 2.1 Synthesis of DFOKC

**Scheme 1:**
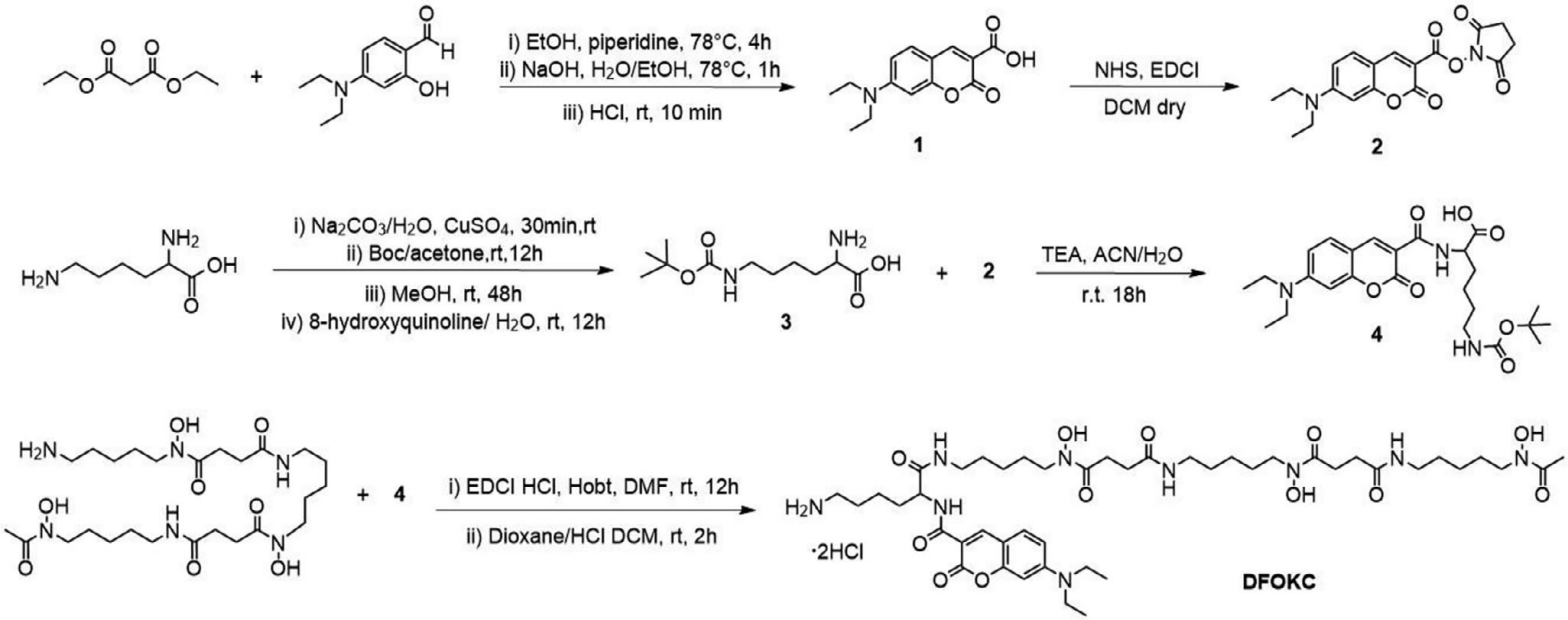
Synthetic pathway of DFOKC.

Synthesis of DFOKC ligand firstly involved the formation of the coumarin scaffold (coumarin 7‐DCCA) via Knoevenagel condensation between 4‐diethylamine salicylaldehyde and diethyl malonate (Scheme 1). The protected derivative of Lys was then synthesized by selectively protecting the ε‐amino group with di‐*tert*‐butyl dicarbonate (Boc) and reducing reactivity of the amine in α position by the formation of a 2:1 Lys‐Cu(II) complex. This complex was then released by using 8‐hydroxyquinoline, yielding the protected primary amine in ε position. N‐hydroxysuccinimidyl ester of coumarin 2 was then reacted with 3 to give product 4. This intermediate was subsequently reacted with DFO mesylate salt by the formation of an amide bond via coupling reaction. The Boc group deprotection step was carried out by adding a 4 M HCl‐dioxane mixture in dry dichloromethane, leading to the precipitation of the final product DFOKC.

### 2.2 Acid Base Properties of the Ligand

**Figure 2:**
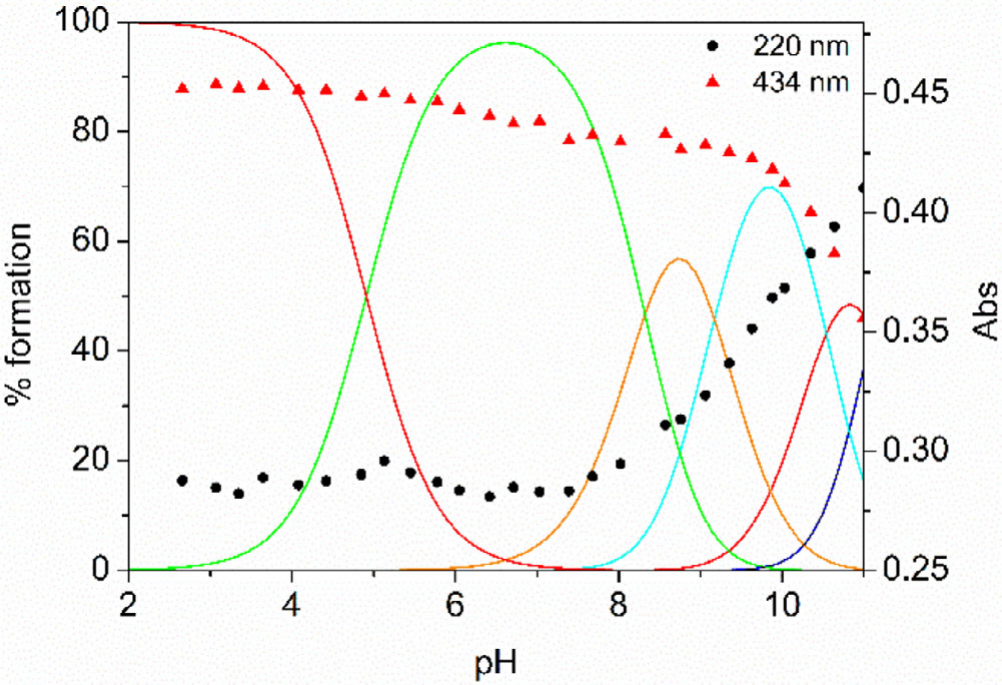
Absorbance values at 220 and 434 nm of DFOKC superimposed to the distribution diagram of the species present in solution (DFOKC is indicated with L, [L] = 10^−5^ M).

As a preliminary step in the study of the DFOKC metal binding properties, we performed UV‐Vis spectrophotometric titrations to determine the protonation constants of the ligand and the corresponding protonated species present in solutions. The ligand in its fully deprotonated form (DFOKC^3−^) displays 5 protonation equilibria, as per results in Table 1. The first 4 protonation equilibria closely resemble the behavior of the pristine DFO ligand. The first protonation constant is ascribed to the primary amine (we remind here the structural difference between DFO and H_3_DFOKC, that is, the nature/position of this amino group is not the same) and the following three to the protonation of the anionic hydroxamate sites, as per the DFO ligand. This confirms a fundamental retention of the desirable basic properties of the DFO ligand. The fifth protonation constant is due to the insertion of the tertiary amine, bore by the coumarin moiety (as observed also for H_3_DFOC).^[^ ^3^ ^]^ According to the above speciation model, the H_4_DFOKC^+^ species, bearing three hydroxamic acid functionalities and a terminal ammonium group, is the main (> 90%) species at physiological pH, as shown in the calculated distribution diagram based on spectroscopic constants (Figure 2).

**Table 1:** Protonation constants of the DFOKC and DFO ligand determined in 0.1 M NMe_4_Cl at 298 K via UV‐Vis spectroscopy. A comparison with literature data for DFO (both in various conditions and in the same ones) is also presented. DFO and DFOKC are indicated with L, making explicit their protonated forms present in solution. Values in parentheses are the standard deviation on the last significant figure.

Equilibrium	log *K* [DFOKC]^[^ ^a^ ^]^	log *K* [DFO]^[^ ^b^ ^]^	log K [DFO]^[^ ^c^ ^]^
L^3−^ + H^+^ = HL^2−^	11.10(7)	10.70 – 11.00	10.70(2)
HL^2−^ + H^+^ = H_2_L^−^	10.54(5)	9.45 – 9.94	9.72(2)
H_2_L^−^ + H^+^ = H_3_L	9.17(5)	8.93 – 9.21	8.93(3)
H_3_L + H^+^ = H_4_L^+^	8.32(6)	8.30 – 8.71	8.35(3)
H_4_L^+^ + H^+^ = H_5_L^2+^	4.91 (5)	–	–

^[a]^ This work, from UV‐Vis spectroscopic measurements.

^[b]^ Literature values range from potentiometric measurements, various ionic strengths.^[^ ^11^ ^]^

^[c]^ From reference 35, obtained via potentiometric measurements in the same conditions of the present work (0.1 M NMe_4_Cl, 298 K).

**Figure 3:**
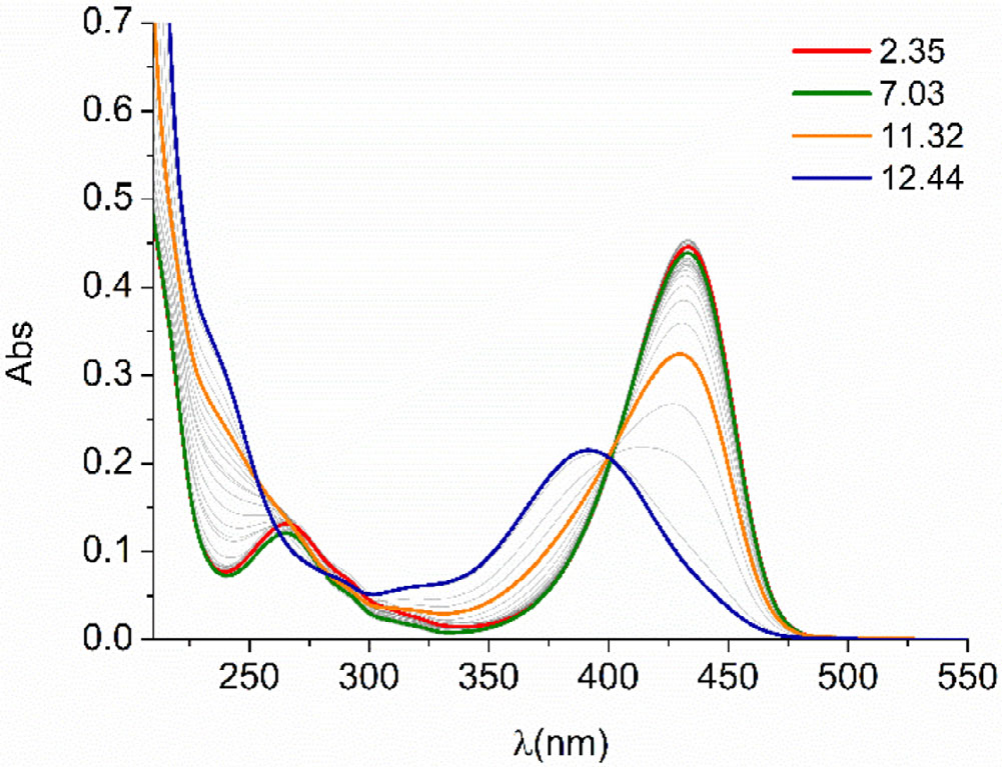
UV‐Vis spectra of DFOKC at different pH values ([DFOKC] = 10^−5^ M, 0.1 M aqueous NMe_4_Cl).

**Figure 4:**
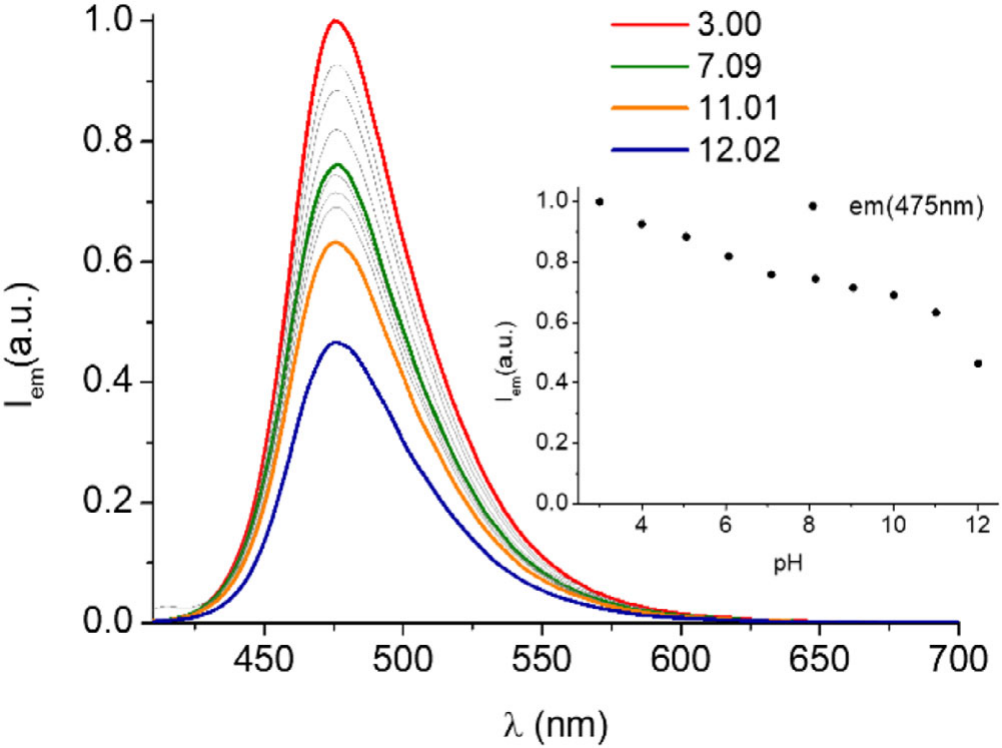
Fluorescence emission spectra of DFOKC at different pH values. Inset: variation of fluorescence emission intensity at 475 nm as a function of pH ([DFOKC] = 10^−5^ M, 0.1 M aqueous NMe4Cl, *λ*_exc_ = 400 nm).

UV‐Vis absorption spectra, reported in Figure 3, are characterized by the presence of the typical pH dependent band related to the hydroxamic groups of DFO, below 250 nm, and lower energy coumarin bands, with the most intense absorptions centered at 434 nm and 265 nm, at least at acidic pH values. The observed spectral changes of the hydroxamate/hydroxamic absorption band below 250 nm strictly resemble the proposed protonation pattern. As expected, no significant variations for this band are observed in acidic or neutral pH conditions, considering that the first protonation equilibrium is attributed to the tertiary amine group of coumarin. On the contrary, an increase in the absorbance can be observed at pH > 8, in correspondence of the deprotonation of the hydroxamic groups. The 434 nm centered coumarin band shows less evident changes in acidic and neutral pH region, with a 6% absorption increase from pH 7 to pH 4. A decrease in the absorbance and a blue shifting of the band of about 40 nm is observed at alkaline pH values (pH > 10), in correspondence of the deprotonation of the primary amine group of the Lys. This behavior can be tentatively justified by hypothesizing hydrogen bonding involving the protonated Lys amine and the CO group of coumarin, or a cation‐π contact with the hetero‐aromatic moiety. These interaction modes could be weakened or lost upon deprotonation of the ammonium group of Lys Furthermore, in the 250–300 nm region, the coumarin absorption at 265 nm experiences a sigmoidal increase of ca 10% from pH 7 to 3 (Figure S5). The latter change, together with the minor variation observed at 434 nm, can be associated with protonation of the coumarin tertiary amine group, for which protonation at acidic pH values has been already observed in methanol/water mixture.^[^ ^44^ ^]^ Fluorescence emission spectra (Figure 4) are characterized by the presence of the typical emission band of coumarin centered at 475 nm upon excitation at 400 nm. The emission intensity undergoes an overall 50% quenching, with a first variation at acidic pH, in correspondence of the deprotonation of the tertiary amine, and a second significant change at alkaline pH, supporting the hypothesis of interaction with the protonated amine group of Lys.

### 2.3 Coordination Properties of the Ligand

**Figure 5:**
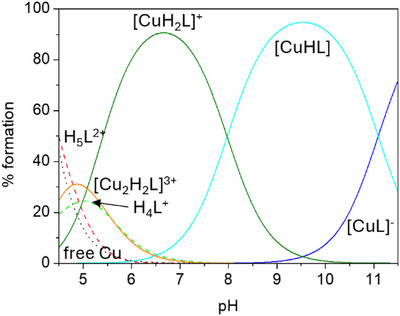
Distribution diagram of the species present in solution of DFOKC in the presence of 1 equivalent of Cu(II) (DFOKC is indicated with L, [L] = [Cu(II)] = 10^−5^ M).

**Figure 6:**
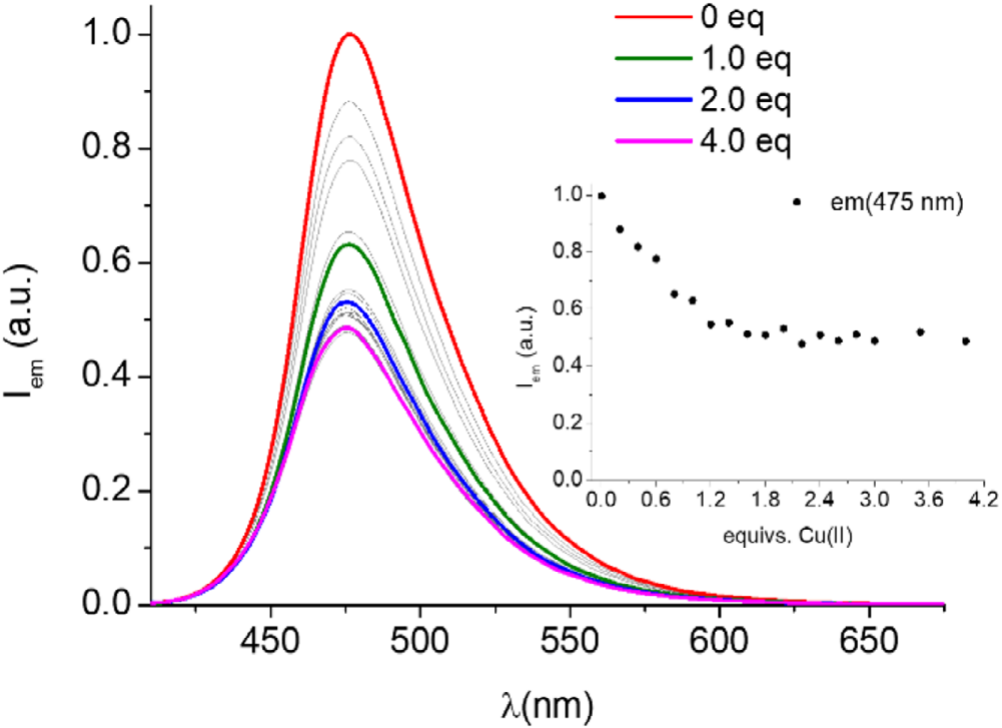
Fluorescence emission spectra of DFOKC in the presence of increasing amounts of Cu(II). Inset: variation of fluorescence emission intensity at 475 nm as a function of Cu(II) equivalents ([DFOKC] = [Cu(II)] = 10^−5^ M, 0.1 M aqueous NMe_4_Cl, *λ*_exc_ = 400 nm).

As previously anticipated, ^64^Cu(II) is one of the first radiometals used in PET imaging, owing to the facts that i) Cu(II) does not generally pose kinetic issues in the coordination of open chain polydentate ligands (among which DFO), nor complications due to its strong hydrolysis (differently from Zr(IV))^[^ ^37^, ^45^, ^46^ ^]^ ii) its coordination by DFO and related ligands has been thoroughly investigated, so that data are available for comparisons^[^ ^11^ ^]^ and iii) as mentioned, copper has useful radioactive nuclides (^64^Cu and ^67^Cu) both for imaging and therapy, making Cu(II) study meaningful in itself. Cu(II) coordination was followed by recording UV‐Vis spectra of DFOKC at different pH values in the presence of 1 and 2 equivs. of metal. This allows for the determination of the stoichiometry of complexes, their stability constants and the distribution of the species present in solution as a function of pH. Figures S9 and S10 show the UV‐Vis spectra of the DFOKC‐Cu(II) 1:1 and 1:2 systems. In the presence of the metal cation, the coumarin band at 343 nm shows significant changes even at weakly acidic pH, unlike the case where there is no metal, where the change occurs only at alkaline pH values. According to DFO data, the study was limited to the 5.0–11.0 pH range (Cu(II) complexation is low below pH 5 due to the competition of H^+^ ions) and extended to include both 1:1 and 2:1 M:L ratios (as DFO is known to form also binuclear complexes with the Cu(II) cations).^[^ ^35^ ^]^ Results are reported in Table 2. As for protonation constants, the ligand is found more basic than pristine DFO. Direct comparison with DFO rather than DFOC is presented in Table 1 as both DFOKC and DFO present a primary amine and a similar protonation pattern (starting from L^3−^ the first proton enters on the amino group), while direct comparison to DFOC, lacking the primary amine function, can be misleading (first proton on DFOC^3−^ enters on an hydroxamic group instead). As a matter of fact, DFOC is already found to form slightly more stable Cu(II) complexes with respect to DFO (Cu(II) + L^3−^ = [CuL]^−^ log K = 14.72 for H_3_DFOC,^[^ ^32^ ^]^ log K = 13.73 for DFO, Table 2). Present data confirms this behavior also for DFOKC. It should be noted that, beyond said difference in protonable sites, both DFOC and DFOKC ligands present, with respect to DFO, extra aliphatic amides and an aromatic ester group: we postulate that these functionalities might assist metal coordination, either directly, as donor atoms (of which we have no explicit evidence), or indirectly, by promoting ligand conformations suitable for metal binding due to the rigid nature of these linkers. Besides above difference, the overall binding properties, including the noncoordinating character of the terminal amine in the 1:1 complex (Cu^2+^ + L^3−^ = [CuL]^−^ and Cu^2+^ + HL^2−^ = [CuHL] have almost equal binding constants for both DFOKC and DFO, Table 2) are coherent with the ones typical of DFO. For what bioassays are concerned, the most representative species at physiological pH is the monocationic [CuH_2_L]^+^ (> 80% formation), although the charge neutral [CuHL] complex is also present (< 20% formation), as shown in Figure 5. Cu(II) coordination also leads to variations in the fluorescence emission properties of the probe. In the presence of the metal cation, DFOKC undergoes a 50% emission quenching upon the addition of 1.5 equivs. of Cu(II) (Figure 6). Successive additions of Cu(II) do not induce further significant changes in the emission intensity. As already observed in the case of DFOC, the distance between the metal‐binding site and the fluorogenic unit prevents the quenching effect upon coordination with the Cu(II) paramagnetic ion.

**Table 2:** Binding constants of the DFOKC and DFO ligand toward Cu(II), determined in 0.1 M NMe_4_Cl at 298 K via UV‐Vis spectroscopy. DFO and DFOKC are indicated with L, making explicit the charged complexes present in solution. Values in parentheses are the standard deviation on the last significant figure.

Species	log *β* [DFOKC]^[^ ^a^ ^]^	log *β* [DFO]^[^ ^b^ ^]^
Cu^2+^ + L^3−^ = [CuL]^−^	15.94(6)	13.73
Cu^2+^ + HL^2−^ = [CuHL]	15.93 (6)	13.14
Cu^2+^ + H_2_L^−^ = [CuH_2_L]^+^	13.36(3)	12.8
Cu^2+^ + [CuHL] = [Cu_2_HL]^2+^	6.61(6)	7.52
Cu^2+^ + [CuH_2_L]^+^ = [Cu_2_H_2_L]^3+^	5.95 (6)	–
2Cu^2+^ + L^3−^ + OH^−^ = [Cu_2_L(OH)]	13.07(7)	–

^[a]^ This work;

^[b]^ potentiometry, 298 K, *I *= 0.2 KCl.^[^ ^19^ ^]^

**Figure 7:**
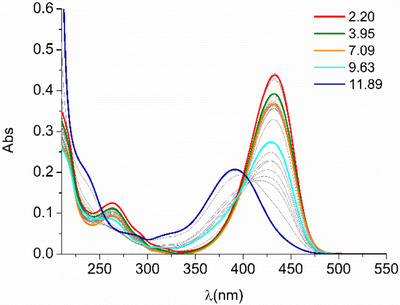
UV‐Vis spectra of DFOKC at different pH values in the presence of 1 equivalent of Zr(IV) ([DFOKC] = [Zr(IV)] = 10^−5^ M, 0.1 M aqueous NMe4Cl).

**Figure 8:**
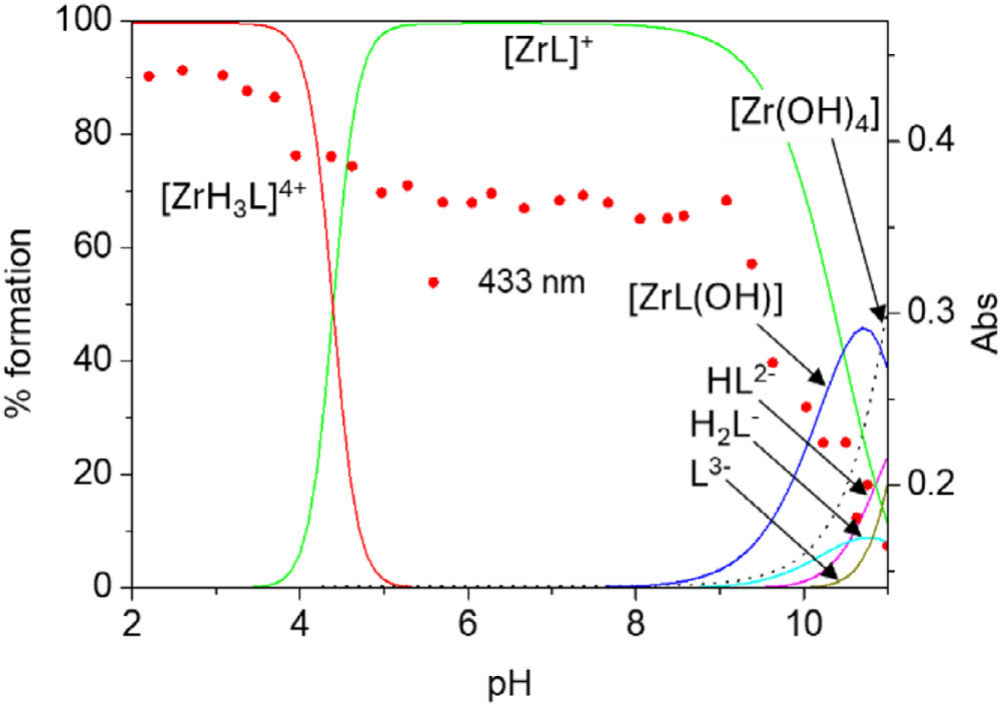
Absorbance values at 265 and 433 nm of DFOKC in the presence of 1 equivalent of Zr(IV) superimposed to the distribution diagram of the species present in solution (DFOKC is indicated with L, making explicit the charged complexes present in solution. [L] = [Zr(IV)] = 10^−5^ M).

**Figure 9:**
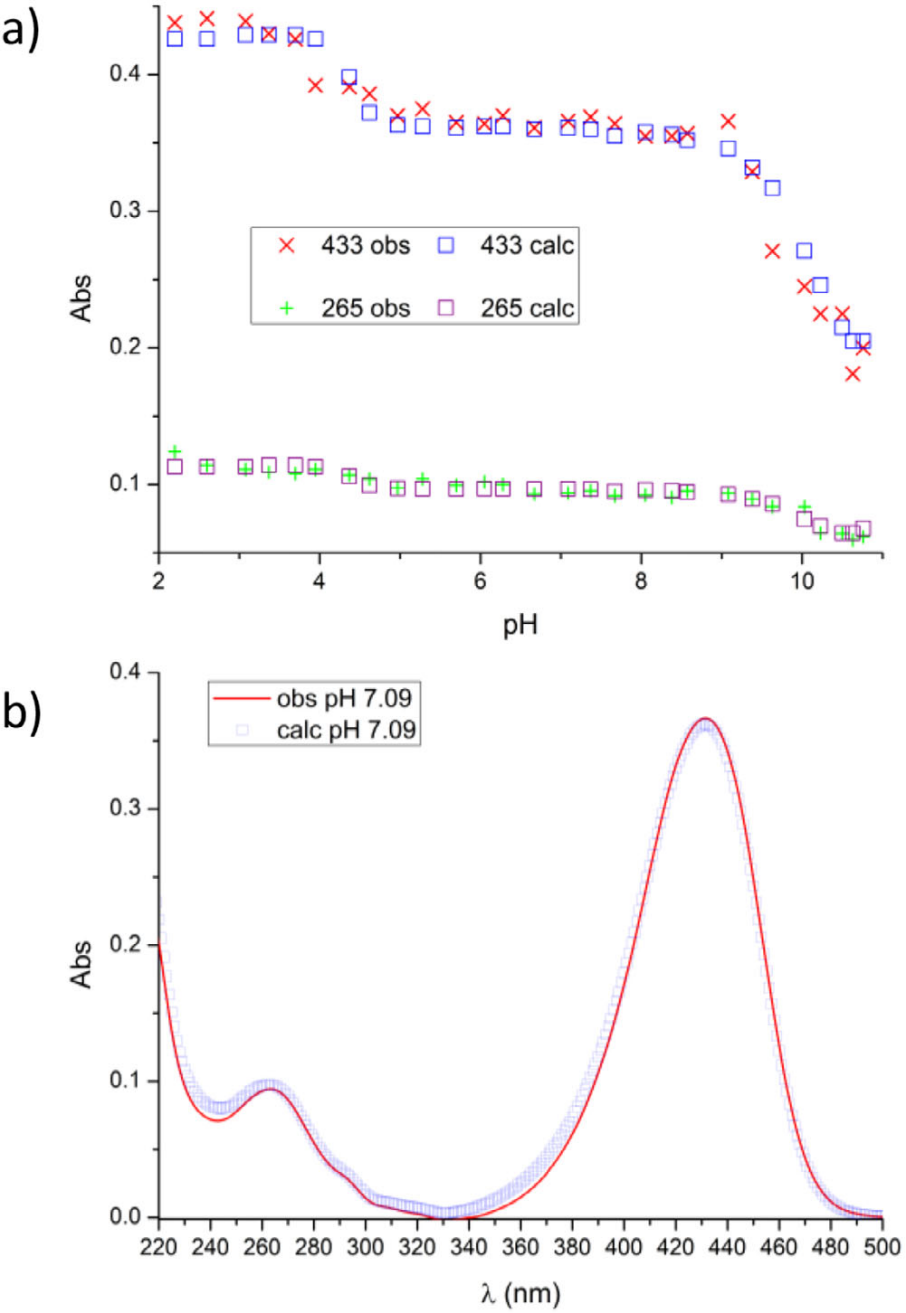
a) Observed (red/green cross) versus calculated (blue/violet squares) absorbances at selected wavelengths as generated by the HypSpec fitting of spectroscopic data for the determination of the Zr(IV) binding constants (reported in Table 3). b) Sample of a whole observed (red line) versus calculated (blue squares) spectrum (pH = 7.09) as resulting from the HypSpec treatment of the ligand's spectra to obtain the set of Zr(IV) binding constants reported in Table 3.

Similar considerations can be made for Zr(IV) binding (Table 3). Also in this case, UV‐Vis absorption spectra recorded at different pH values in the presence of 1 equiv. of Zr(IV) (Figure 7) were used to determine the species present in solution (Figure 8) and their formation constants (HypSpec fitting of spectroscopic data is shown in Figure 9). The proposed speciation model is simpler with Zr(IV) than with Cu(II), although we must be wary of the peculiarity of the employed technique for binding constant determination (UV‐Vis spectroscopy). As known and further detailed in the work of Romano et al., hydroxamate/hydroxamic acid equilibrium affects the UV bands of the ligand.^[^ ^32^ ^]^ As metalation mimics protonation (in terms of electronic doublet availability), it is clear that the spectrum of the free ligand in the alkaline range (HL^2−^ and L^3−^ as dominant species, both bearing 3 hydroxamate anionic sites) is significantly altered in the presence of Zr(IV), as the metal binds to available hydroxamate sites. The ZrL^+^ complex, thus appears to dominate from pH 4 to pH 10, appearing as the only formed species in said pH range. The ZrHL^2+^ complexes is likely not observed as the protonation of the ammonium group is rather spectroscopically silent. This is not the case for the softer (compared to Zr(IV)) Cu(II) cation, as the amine/ammonium equilibrium can alter optical properties (at least under metal excess conditions, that is, for 1:2 L:Cu(II) complexes).

**Table 3:** Binding constants of the DFOKC ligand toward Zr(IV), determined in 0.1 M NMe_4_Cl at 298 K via UV‐Vis spectroscopy. Values in parentheses are the standard deviation on the last significant figure.

Species	log *β* (DFOKC)
Zr^4+^ + L^3−^ = [ZrL]^+^	37.58(2)
Zr^4+^ + H_3_L = [ZrH_3_L]^4+^	24.21(4)^*^
[ZrL]^+^ + OH^−^ = [ZrL(OH)]	3.36 (2)

* Assuming the ligand has 2 protonated amines (primary and tertiary) and only one protonated noncoordinating hydroxamic acid group

Detachment of a single hydroxamate group from Zr(IV) and its protonation is also a known process, both for DFO,^[^ ^35^, ^36^, ^37^ ^]^ H_3_DFOC,^[^ ^32^ ^]^ and other analogues.^[^ ^46^ ^]^ Still distinguishing (from the UV‐Vis spectrum) a metal coordinate hydroxamate from a hydroxamic acid can be untrivial. In the case of DFOKC the more so, as the spectral variation in the acidic range are dominated by the formation of the ZrH_3_L^4+^ species. As the system reaches 50:50 distribution between ZrH_3_L^4+^ and ZrL^+^ at pH 4.4 (protonation of the secondary amine of the nonmetalated ligand has a log K of 4.91(5), Table 1), and the observed spectral variations are large and coherent with above proposed protonation scheme, this third proton enters on the fluorophore tertiary amine.

**Figure 10:**
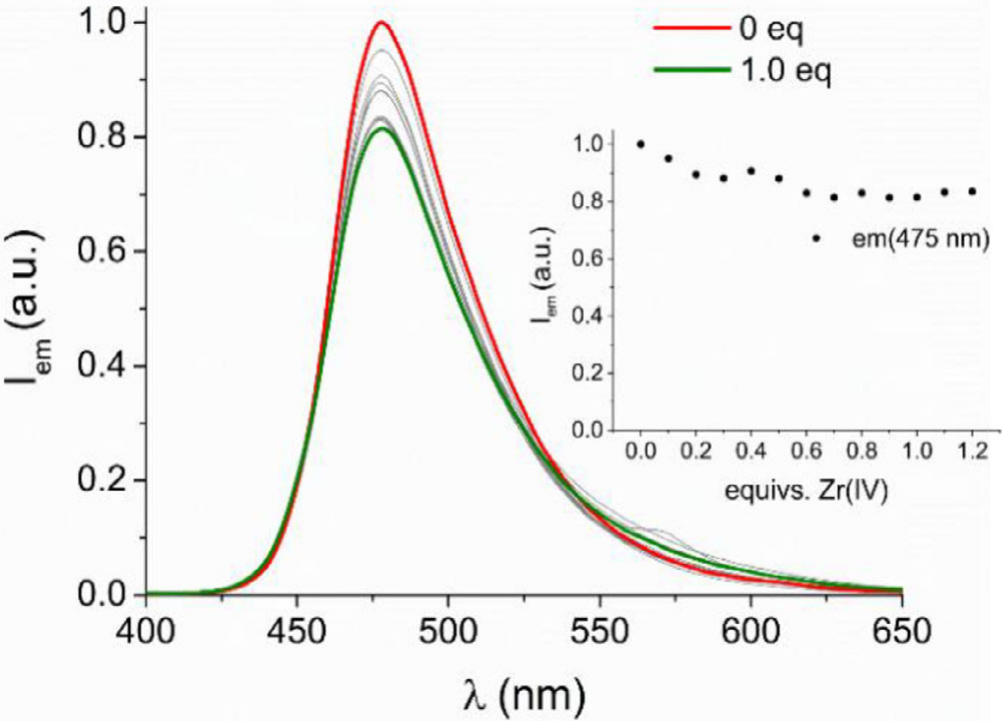
Fluorescence emission spectra of DFOKC in the presence of increasing amounts of Zr(IV). Inset: variation of fluorescence emission intensity at 475 nm as a function of Zr(IV) equivalents ([DFOKC] = [Zr(IV)] = 10^−5^ M, 0.1 M aqueous NMe_4_Cl, *λ*_exc_ = 400 nm).

Thus, only ZrL^+^ and ZrH_3_L^4+^ are clearly detected species due to their significantly different spectral properties, although some of the protonated species in between are also likely to actually exist in the real system. As the primary amino group has high protonation constant (log K > 11) and there is consensus that it does not take part in Zr(IV) coordination, we expect the ZrHL^2+^ complex to be the most representative species at physiological pH, despite the fact that its formation constant could not be determined from spectroscopic data. Lastly, as for both DFO^[^ ^35^, ^36^, ^37^ ^]^ and DFOC,^[^ ^32^ ^]^ a monohydroxo‐species can be formed in alkaline medium above pH 9.5, giving rise to significant spectral changes. As a meaningful simple comparison, the Zr^4+^ + L^3−^ = [ZrL]^+^ equilibrium has a reported log K of 36.02(9) for DFO, 39.3(1) for DFOC, and 37.58(2) for DFOKC: both derivatives appear slightly more basic than pristine DFO (according to both the trend seen for their basicity and Cu(II)‐binding constants). Overall, we can conclude that the favorable binding properties of pristine DFO toward Zr(IV) are maintained, if not enhanced, in H_3_DFOKC. As already mentioned for DFOC,^[^ ^32^ ^]^ Zr(IV) complexation exhibits minimal interference with fluorescence emission (see Figure 10). When increasing amounts of Zr(IV) are added to DFOKC solution at pH 7, where single species ZrL^+^ is present, a steady fluorescence quenching is observed, though the decreasing does not exceed 20%. This modest effect can be attributed to the distancing between the fluorophore and the binding group, that limits the quenching effect of heavy metal coordination. Nevertheless, the Zr‐DFOKC complex remains sufficiently emissive to be used in optical imaging techniques for biological studies.

### 2.4 In Vitro Cell Tolerability Studies

**Figure 11:**
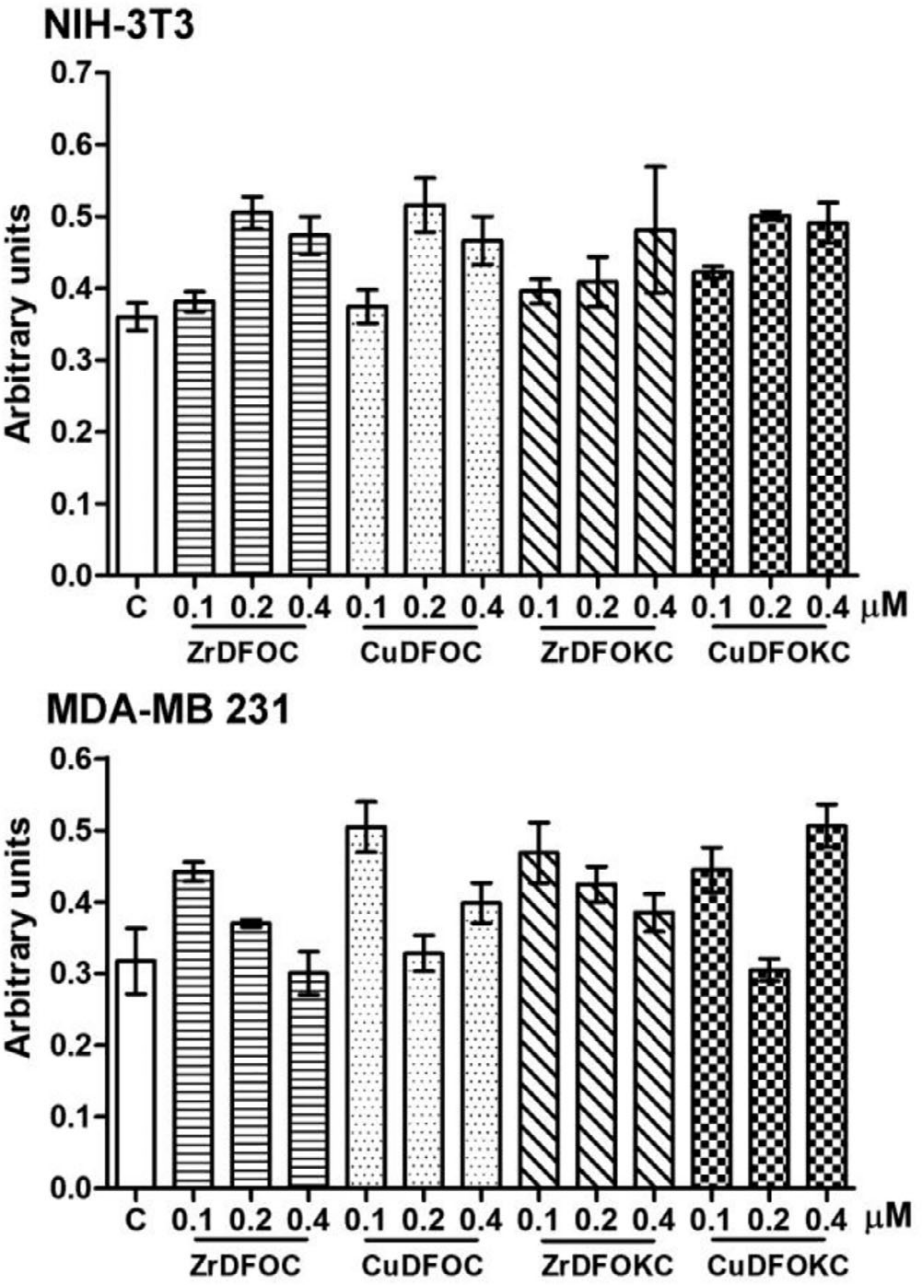
Cell viability assay by crystal violet vital dye uptake on NIH‐3T33 fibroblasts and MDA‐MB 231 human breast adenocarcinoma cells exposed to the noted compounds for 24 hours at increasing concentrations. No significant decrease in cell viability was detected at any concentrations assayed. C, control. Bars are the mean ± SEM of 3 replicate experiments. (one‐way ANOVA and Newman Keuls post‐test).

**Figure 12:**
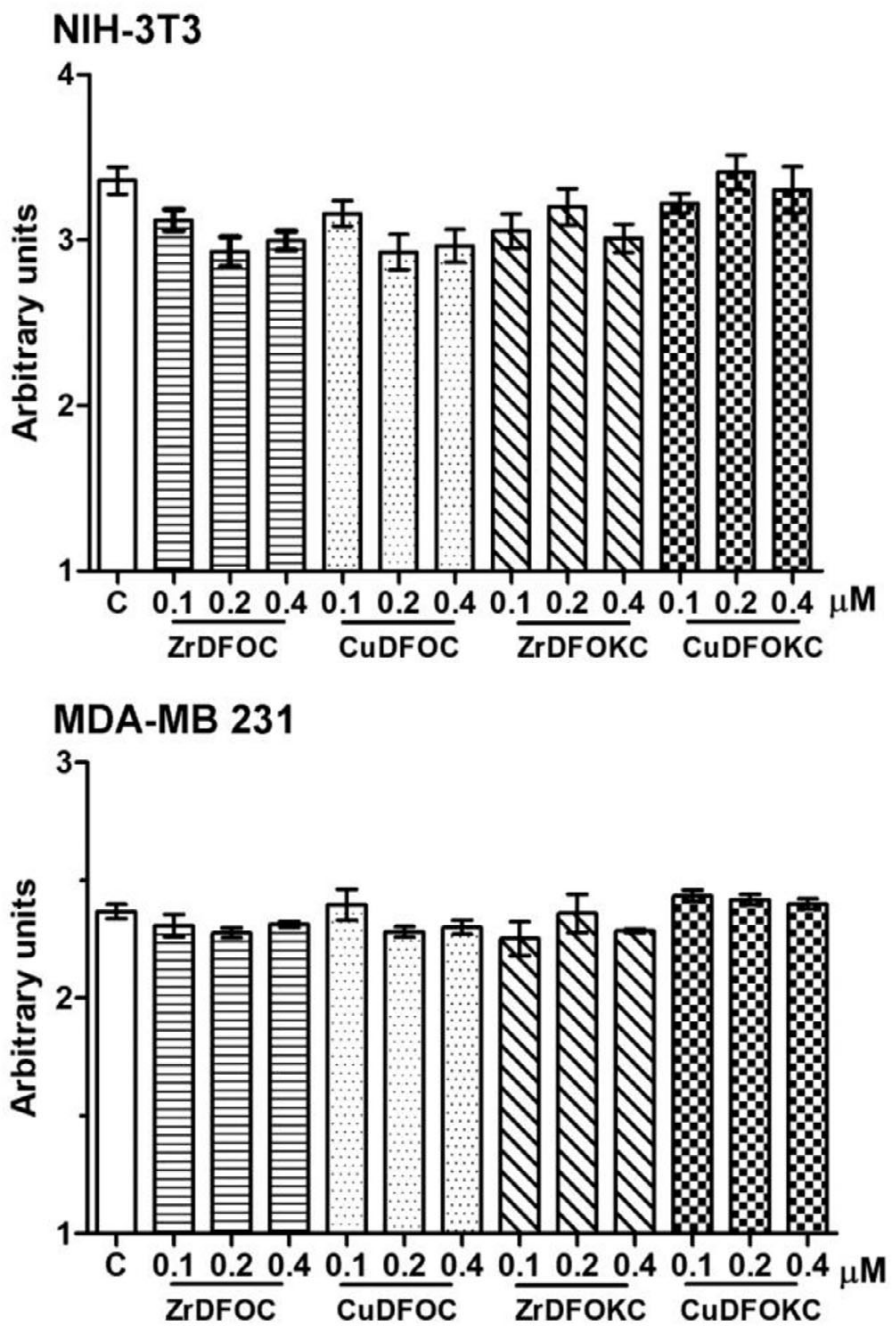
MTT mitochondrial metabolism assay on NIH‐3T3 fibroblasts and MDA‐MB 231 human breast adenocarcinoma cells exposed to the noted compounds for 24 hours at increasing concentrations. No significant changes in mitochondrial respiratory metabolism were detected. C, control. Bars are the mean ± SEM of 3 replicate experiments. (one‐way ANOVA and Newman Keuls post‐test).

The effects of the compounds under study on cell viability (crystal violet assay) and mitochondrial respiratory function (MTT assay, MTT = 3‐[4,5‐dimethylthiazol‐2‐yl]‐2,5 diphenyl tetrazolium bromide) were evaluated on both NIH‐3T3 normal fibroblasts and MDA‐MB 231 mammary adenocarcinoma cells. These experiments’ results indicate that none induced cytotoxic effects or mitochondrial impairment. This was not only observed with the lower 0.1 µM concentration assayed, similar to that commonly used in clinical practice for most radio‐diagnostics,^[^ ^45^ ^]^ but also with 0.2 and 0.4 µM (Figures 11 and 12). This finding is in keeping with their high chemical stability, making it unlikely that the potentially toxic metal center may be released in the normal cellular environment.

### 2.5 In Vitro Biodistribution Studies

**Figure 13:**
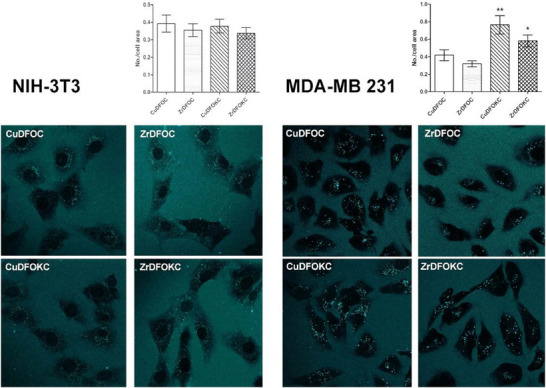
Fluorescent (*λ* 540 nm emission) images of NIH‐3T3 fibroblasts and MDA‐MB 231 breast adenocarcinoma cells exposed to the noted compounds (0.1 µM) for 10 minutes. A dotted cyan fluorescence can be seen within the cells. A faint, diffuse cyan fluorescence can also be seen in the extracellular medium. Confocal microscopy, magnification ×630. The bar graphs show the number of fluorescent dots per cell area: NIH‐3T3 fibroblasts show no statistically significant differences among the tested compounds, whereas MDA‐MB 231 cancer cells show a significant increase in the cells treated with both Cu‐ and Zr‐DFOKC as compared with the parental compounds Cu‐ and Zr‐DFOC (*p < 0.05, **p < 0.01; one‐way ANOVA and Newman Keuls post‐test).

NIH‐3T3 fibroblasts and MDA‐MB 231 mammary adenocarcinoma cells were incubated for 10 minutes in Dulbecco's Modified Eagle Medium (DMEM) supplemented with each compound (0.1 µM) to study their distribution in the extra‐ and intracellular compartments by confocal microscopy, exploiting the *λ* = 405–540 nm excitation‐emission pair of their coumarin moiety as tracking signal (cyan). A slight, diffuse cyan fluorescence was detected in the extracellular medium, while an intense, dotted fluorescent signal was visible within the cytoplasm, suggesting that endocytosis of the compound had occurred (Figure 13). This effect was more prominent in the neoplastic than in the normal cells, likely because of the higher metabolic activity in the former. Moreover, in the MDA‐MB 231, CuDFOKC induced the most prominent stimulation of endocytosis, reaching statistical significance in comparison with the other compounds.

**Figure 14:**
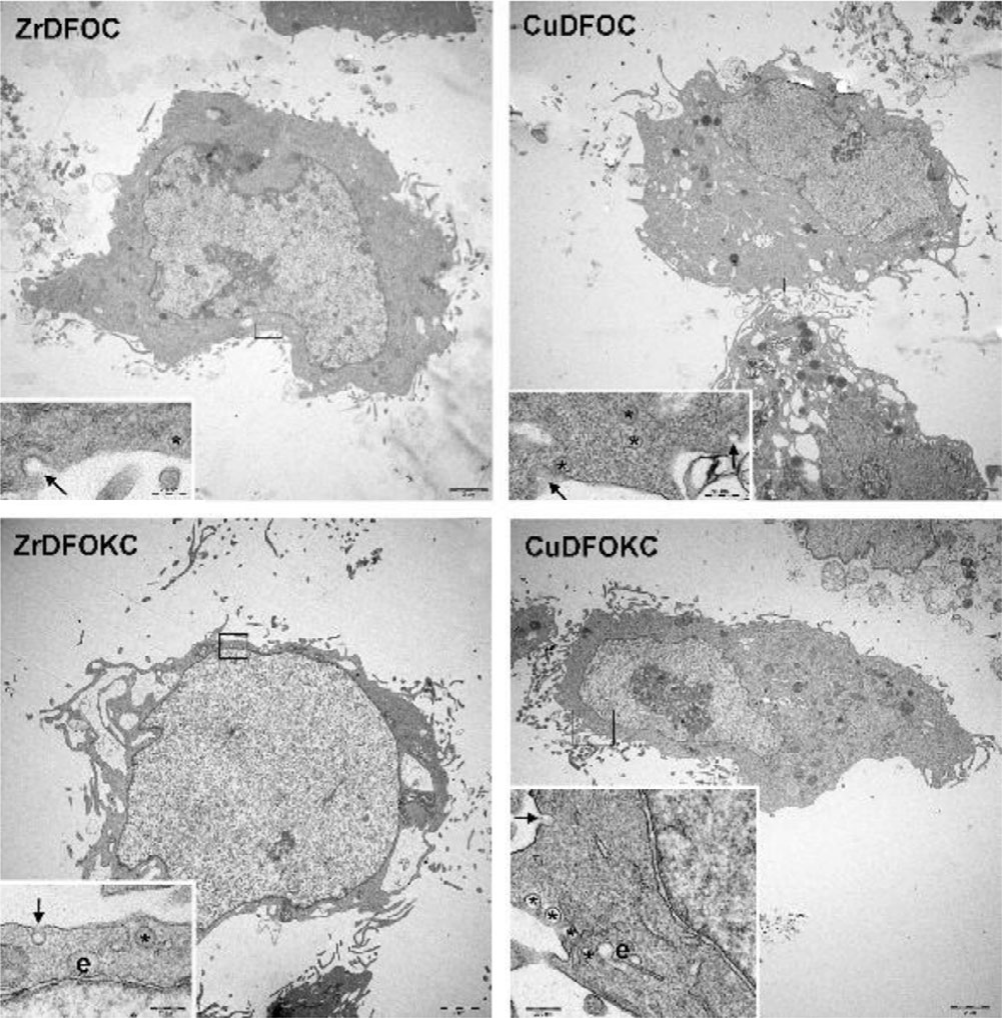
Representative ultrastructural images of MDA‐MB 231 human breast adenocarcinoma cells treated with the noted compounds (0.1 µM) for 10 minutes. The cells show a normal organelle complement with no signs of damage. Plasma membrane pits (arrows) and microvesicles in the peripheral cytoplasm (asterisks) are suggestive of pinocytosis. Some vesicles appear to fuse with cortical endosomes (e). The images of pinocytosis were more frequently observed in the cells exposed to CuDFOKC. Magnification is indicated under the bars.

To confirm the above observations, MDA‐MB 231 human breast adenocarcinoma cells treated for 10 minutes. with each compound (0.1 µM) were examined by the electron microscope. These cells showed a well‐developed organellular complement with normal features and no signs of damage. Plasma membrane pits and peripheral micro‐vesicles, suggesting the occurrence of pinocytosis, were often observed (Figure 14).

Taken together, the above findings suggest that the presence of additional amino groups in the Cu‐ and ZrDFOKC compounds induces a higher solubility in the extra‐ and intra‐cellular watery milieu and enhanced capability to enter the cells, conceivably through pinocytosis. This is likely due to the presence at neutral pH of protonated amine group, which can strongly interact via charge–charge and hydrogen bonding contacts with the carboxylate groups featuring the cell membrane.

### 2.6 In Vitro Radiosensitivity Studies

**Figure 15:**
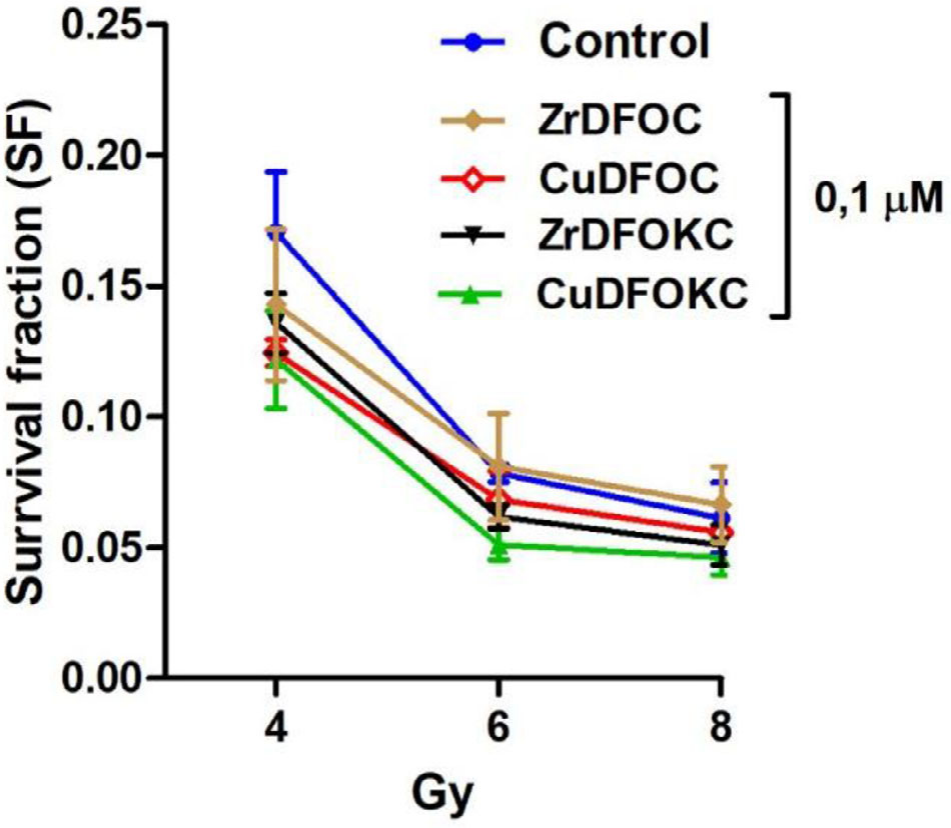
Survival curves calculated from clonogenic assay of MDA‐MB 231 human breast adenocarcinoma cells treated or not with the noted compounds (0.1 µM) and exposed to X‐irradiation at increasing doses. (Two‐way ANOVA: *p* = 0.62, not significant).

Given the possible use of the compounds under study to develop new photo‐radiopharmaceuticals, we next aimed to assess whether they would interfere with the sensitivity of neoplastic cells to X‐radiation therapy. Ionizing radiations induce cell death by both direct effects due to DNA strand breaks and indirect effects mediated by increased generation of reactive oxygen species (ROS) which, in turn, cause oxidative damage to nucleic acids, proteins, and membrane lipids.^[^ ^47^ ^]^ In conditions of oxidative stress, some metal chelates, for instance those containing Cu(II), can reduce ROS levels, because the metal center can scavenge the ROS radicals through Cu(II)/Cu(I) redox cycles, thus catalyzing ROS transformation into inert chemical species.^[^ ^48^ ^]^ On the other hand, several molecules and metal compounds are known to act as radiosensitizers, being able to potentiate the cellular mechanisms of susceptibility to radiations. In fact, in some radiotherapy protocols, these molecules are used to reduce the radiation dose.^[^ ^49^ ^]^ In the present experiment, a clonogenic (i.e., colony‐forming) assay was used to evaluate whether the radiosensitivity of MDA‐MB 231 mammary adenocarcinoma cells was influenced by the addition of the compounds under study, following a protocol previously used for similar purposes.^[^ ^32^ ^]^ As shown in Figure, no significant differences were detected between the untreated control cells and those incubated with either compound, indicating that they did not interfere, either positively or negatively, with the cytotoxic effects of increasing X‐ray doses (4‐8 Gy), like those used in clinical radiotherapy (Figure 15).

## 3 Conclusions

Compared to DFOC, the insertion of a fluorescent coumarin unit by using a Lys‐based linker restores many of the key favorable properties of the DFO parent molecule, one of the most used chelating agents in PET imaging, which were partially lost in DFOC. While ligand speciation and protonation pattern were significantly affected in moving from DFO to DFOC, restoring the primary amine groups allows for a virtually identical speciation/overall charge (besides the fluorophore amino group protonation below pH 5) between DFO and DFOKC; this, in turn, allows for more meaningful comparison toward the metal binding abilities of the two ligands. Stability and stoichiometry of Cu(II) and Zr(IV) complexes are effectively maintained, moreover with some stability improvements, in comparison to the golden standard chelator DFO. The restoration of the primary amine appears of high relevance also from a synthetic and bioconjugation viewpoint, making DFOKC suitable for further derivatization and, most likely, amenable to conjugation protocols similar to the established ones for DFO. These features were absent in DFOC as, in that case, the primary amine was sacrificed in the conjugation with the fluorophore and thus no longer available for further synthetic modifications. Conversely, DFOKC maintains some attractive characteristics of DFOC. In fact, it shows useful fluorescence emission which is essentially not quenched by Cu(II) and Zr(IV) coordination. A further striking result of this work is represented by the increased uptake of its complexes by cells: compared to DFOC the presence of additional amino group in the DFOKC scaffold, protonated at physiological pH in both Cu(II) and Zr(IV) complexes, reinforce their interaction with the negative carboxylate groups of sialic acid located at the plasma membrane's outer oligosaccharide layer, favoring cellular uptake and internalization through pinocytosis. Furthermore, as for DFO and DFOC analogues, no adverse effects on cell respiratory metabolism and viability were observed.

Giving the lack of chemical and cellular adverse effects, we can conclude the reintroduction of the terminal amino group represents a viable strategy for further addition of targeting vectors, such as biotin, folate, or tumor‐specific mAbs, capable of enhancing the tropism of these compounds toward specific target cells, especially neoplastic cells.^[^ ^50^, ^51^, ^52^, ^53^ ^]^

## 4 Experimental Section

### Spectroscopic measurements

Absorption spectra were recorded at 298 K on a Jasco V‐670 spectrophotometer (Jasco Europe, Lecco, Italy). For the UV‐Vis spectra, the ligand concentration was 10 µM in all experiments, 10 µM for metals cations (Cu(II) or Zr(IV)) in 1:1 experiments, and 20 µM for DFOC:Cu(II) 1:2 titrations. In 1:1 Zr(IV)‐ligand UV‐Vis titration, 29 samples were prepared. In each sample, the pH was adjusted to the exact value, and they were kept under stirring overnight to reach equilibrium.

Fluorescence spectra were recorded at 298 K on a FluoroMax Plus spectrofluorometer (HORIBA France, Longjumeau Cedex, France). In fluorescence experiments, pH titrations were conducted in solutions where the ligand concentration was 10 µM, and titrations with metal cations were performed in solutions where the ligand concentration was 1 µM.

### Refinement of the stability constants

Protonation constants of DFOKC and binding constants of Cu(II) and Zr(IV) complexes were obtained by treating UV‐Vis spectroscopic data using the HypSpec program.^[^ ^54^ ^]^ The software allows to simultaneously use any number of wavelengths for the least‐squares minimization. The whole 220–500 nm range (280 wavelengths) was used for the fitting of ligand absorbance data. Various models were tested until convergence was reached. As an output, both the model presented in table and the simulated spectra of each ligand species were obtained. These data were used as fixed parameters in the determination of metal binding constants.

### Preparation of H_3_DFOKC complexes stock solution for bioassay

Solution of Cu‐DFOC has been prepared by dissolving 1.9 mg di DFOC in 50.4 mL of DMSO 10% aqueous solution, and 1 equiv. of Cu(II) maintaining pH around neutral range. The sample was left stirring overnight. After adjusting pH at 6.5 the solution was filtered with a filter of 0.45 µm. The final concentration after filtration was measured by UV‐Vis spectroscopy, yielding a value of di 1⋅10^−5^ M.

Solution of Cu‐DFOKC has been prepared by dissolving 3.7 mg di DFOKC in 25.0 mL of DMSO 5% aqueous solution, and 1 equiv. of Cu(II) maintaining pH around neutral range. The sample was left stirring overnight. After adjusting pH at 6.5 the solution was filtered with a filter of 0.45 µm. The final concentration after filtration was measured by UV‐Vis spectroscopy, yielding a value of di 1.28·10^−4^ M.

Solution of Zr‐DFOKC has been prepared by dissolving 4.0 mg di DFOKC in 25.1 mL of DMSO 5% aqueous solution, and 1 equiv. of Zr(IV), maintaining pH around neutral range. The sample was left stirring overnight. After adjusting pH at 6.9 the solution was filtered with a filter of 0.45 µm. The final concentration after filtration was measured by UV‐Vis spectroscopy, yielding a value of di 1.25⋅10^−4^ M.

### In vitro cell cultures

The NIH‐3T3 normal mouse fibroblast line and MDA‐MB 231 human mammary adenocarcinoma line (both from American Type Culture Collection ATCC, Manassas, VA, https://www.atcc.org) were chosen as representative of normal and neoplastic cells, respectively. The cells were grown in DMEM added with 10% foetal bovine serum, 2 mM glutamine, 250 U/ml penicillin G, and 250 µg/ml streptomycin, kept at 37 °C in a humidified air atmosphere with 5% CO_2_. The possible toxic effects of the CuDFOKC and ZrDFOKC compounds, compared with the parental molecule CuDFOC. Data on ZrDFOC were reported in our previous publication^[^ ^32^ ^]^ (unless otherwise stated, the experiments were carried out in triplicate).

### Cell viability assay

The effects of the noted compounds on the viability of NIH‐3T3 fibroblasts and MDA‐MB 231 mammary adenocarcinoma cells were checked by the crystal violet assay. Each compound was added to the culture medium at 0.1, 0.2, and 0.4 µM final concentrations for 24 hours. After that, the medium was removed and the cells were stained with crystal violet vital dye (1% in 70% aqueous ethanol, 15 minutes.), then washed carefully to remove the unbound dye, dried, and dissolved in 10% aqueous acetic acid. The optical density of the violet‐colored solution, directly related to the number of viable cells, was measured with a multiplate spectrophotometer (Bio‐Rad 550, Milan, Italy) at *λ* = 595 nm.

### Mitochondrial metabolism assay

The effects of the noted compounds on mitochondrial energy metabolism of NIH‐3T3 fibroblasts and MDA‐MB 231 mammary adenocarcinoma cells were checked by the MTT assay (MTT = 3‐[4,5‐dimethylthiazol‐2‐yl]‐2,5 diphenyl tetrazolium bromide). Each compound was added to the culture medium at 0.1, 0.2, and 0.4 µM final concentrations for 24 hours. Then, the cultures were added with MTT, a tetrazolium salt reduced to insoluble formazan by mitochondrial enzymes. The formazan crystals were then dissolved in dimethylsulfoxide and the optical density of this purple‐colored solution, directly related to mitochondrial respiratory function, was read at the spectrophotometer (Bio‐Rad) at *λ* = 540 nm.

### Biodistribution assay

The distribution of the compounds under study in the extra‐and intra‐cellular microenvironments of normal NIH‐3T3 and neoplastic MDA‐MB 231 cells were studied by confocal microscopy, exploiting the fluorescent signal of the coumarin moiety. Cells grown on glass coverslips were treated with each compound added to the culture medium at a 0.1 µM final concentration for 10 minutes. Then, the coverslip was mounted over a glass slide and observed at a Leica Stellaris 5 (Leica Microsystems, Milan, Italy) confocal microscope equipped with a *λ* 405 nm excitation laser, setting the spectral sensor at *λ* 500 nm coincident with the coumarin emission peak, and using a ×63 oil immersion objective. Quantitative analysis of the number of fluorescent pinocytosis vesicles per cell surface area was performed using the free‐share Fiji‐ImageJ 1.54f image analysis software (http://imagej.org).

### Transmission electron microscopy

The MDA‐MB 231 mammary adenocarcinoma cells were also studied ultrastructurally to collect additional data on cytotoxicity and cellular uptake of the studied compounds. Each compound was added to the culture medium at a 0.1 µM final concentration for 10 minutes, then the cells were detached from the culture plates with a scraper, pelleted by centrifugation at 600 g, fixed in Karnowsky's fluid (3% glutaraldehyde and 2% formaldehyde in 0.1 M phosphate buffer, pH 7.4), post‐fixed in 1% OsO_4_ in 0.1 M phosphate buffer, pH 7.4, and embedded in epoxy resin. Ultra‐thin sections were cut ad observed at a JEM‐1010 transmission electron microscope (Jeol, Tokyo, Japan).

### Clonogenic survival assay

MDA‐MB 231 mammary adenocarcinoma cells were seeded into 6‐well plates at four low densities to achieve singly dispersed cells and incubated in DMEM added or not with each compound under study (0.1 µM). After 1 hour, a single dose of 0, 4, 6, or 8 Gy of X‐irradiation was delivered by a linear accelerator (Elekta Versa HD^©^), set to deliver an exact, homogeneous dose at the level of the cell layers. For each dose, the wells were subdivided into two groups, with or without compound, as summarized in Table 4. After 4 hours, the medium was replaced with fresh DMEM, and the cells were left to grow and checked daily until the surviving cells proliferated to give rise to individual colonies, 30–50 cells each. At this time (day 6 post‐irradiation), the medium was removed, the cultures were washed in PBS, fixed in 4% formaldehyde in PBS, and stained with crystal violet, as described above. Using a digital video camera interfaced with a computer, live images of each plate at a ×500 magnification were viewed on the screen and distinct colonies made up of no less than 30 cells were counted in a test area of about 5 cm^2^. The surviving fraction (SF) was calculated by the formula: SF = (mean colony count/seeded cells) x plating efficiency (PE), where PE = mean colony count/cells seeded for unirradiated controls. PE was normalized to the value of the respective control for each experiment.^[^ ^55^ ^]^ The SFs were compared statistically by two‐way ANOVA.

**Table 4:** Cell density at seeding depending on radiation doses.

Cells/Well	X‐ray dose [Gy]
750	0 (control)
2000	4
2500	6
3000	9

## Conflict of Interest

The authors declare no conflict of interest.

## Supporting information



Supporting Information

## Data Availability

The data that support the findings of this study are available in the supplementary material of this article.
