# Supplementary material for: Fluorescent Deferoxamine Complexes of Cu(II) and Zr(IV): Insights in the Development of Dual Imaging Probes
Source: Chemistry. 2025 May 27;31(35):e202501203. doi: 10.1002/chem.202501203 (PMC12188159; doi:10.1002/chem.202501203)
Supplement: Supplementary file 1 — Supporting Information [file CHEM-31-e202501203-s001.pdf]

Supporting Information

Fluorescent Desferoxamine Complexes of Cu(II) and Zr(IV): Insights in the Development of Dual Imaging Probes

Yschtar Tecla Simonini Steiner,[a] Virginia Zizi,[b] Monica Mangoni,[c] Patrizia Nardini,[b] Daniele Bani,*[b] Andrea Bencini,*[a] Antonio Bianchi,[a] Matteo Savastano,*[d] Giammarco Maria Romano[a]

[a] Department of Chemistry "Ugo Schiff", University of Florence, Via della Lastruccia 3, 50019 Sesto Fiorentino, Italy

[b] Imaging Platform, Department of Experimental & Clinical Medicine, University of Florence, Viale G.Pieraccini 6, 50139 Florence, Italy

[c] Radiotherapy Unit, Department of Experimental & Clinical Biomedical Sciences, University of Florence, Largo Brambilla 3, 50134 Florence, Italy

[d] Department for the Promotion of Human Science and Quality of Life, University San Raffaele Roma, Via di Val Cannuta 247, 00166 Rome, Italy

Table of Contents

Synthetic procedures **S2**

7-(Diethylamino)coumarin-3-carboxylic acid (1) S2

7-(Diethylamino)coumarin-3-carboxylic acid N-succinimidyl ester (2) S2

2-amino-6-((tert-butoxycarbonyl)amino)hexanoic acid (3) S2

6-((tert-Butoxycarbonyl)amino)-2-(7-(diethylamino)-2-oxo-2H-chromene-3-carboxamido)hexanoic acid (4) S2

H_3DFOKC S3

NMR and ESI MS characterization **S4**

UV-Vis spectroscopic data **S6**
